# Student perception data on online learning using heutagogy approach in the Faculty of Mathematics and Natural Sciences of Universitas Negeri Makassar, Indonesia

**DOI:** 10.1016/j.dib.2020.105152

**Published:** 2020-01-21

**Authors:** R. Rusli, Abdul Rahman, Helmi Abdullah

**Affiliations:** a Department of Mathematics, Universitas Negeri Makassar, Indonesia; b Department of Physics, Universitas Negeri Makassar, Indonesia

**Keywords:** Heutagogy, Online learning, Industrial revolution 4.0, Learning process

## Abstract

One of the important things in the Industrial Revolution 4.0 era was online learning. Therefore, this article describes data about student perceptions related to online learning and data on how long students have been familiar with the internet, what devices are used, and what they are used for. This data includes all departments in the Faculty of Mathematics and Natural Sciences of Universitas Negeri Makassar (FMIPA UNM), Indonesia. The sample in this data are 250 students from various departments at FMIPA UNM. The data collection method used was a questionnaire. The results of this data collection we present some of the questions variables/items included in the dataset. The findings from the data show that about 75% of students of FMIPA UNM agree with the use of online learning. In general, the characteristics of students in every province in Indonesia are “similar” in terms of thinking related to online learning i.e. millennial students, moreover most students already use the internet as a primary need for learning, so this data has policy implications for higher education in Indonesia during the industrial revolution 4.0.

**Table:** Specifications Table

Subject	Social Science
Specific subject area	Education “Online Learning”
Type of data	Table and Figure
How data were acquired	The data in this article was obtained from 250 students from various departments at FMIPA UNM. The participants filled out the questionnaire.
Data format	Raw and Analyzed
Parameters for data collection	The data collected is data related to students' perceptions about online learning. This data includes: how long have you known the internet, how long to surf in a day, opinions about online learning: do they agree with the use of online learning media, do they agree with the heutagogy learning method, does online learning benefit them.
Description of data collection	Data was collected using a questionnaire method that was shared online by matching data with the campus academic information system. If there is data entry that is incompatible with student data in the academic information system, then the data will not be used and is included in the missing data category. This questionnaire was distributed to all departments in FMIPA UNM, namely 7 departments.
Data source location	Population sample data provided in this article was obtained at the Faculty of Mathematics and Natural Sciences, Universitas Negeri Makassar, Makassar City, Republic of Indonesia, (latitude: 5°11′10.5396″ S and longitude: 119°25′37.4268″ E), and with google maps: https://goo.gl/maps/655nZTYUf4srKife7.
Data accessibility	The data available in Mendeley Data: https://doi.org/10.17632/8myyf5t45z.3 Or Direct URL to data: https://data.mendeley.com/datasets/8myyf5t45z/3

**Table:** 

**Value of the Data** • This data is useful for providing information to policy makers in Higher Education especially at Universitas Negeri Makassar on the importance of using online learning in the industrial revolution 4.0. The characteristics of the data (students) have almost the same similarity with students in general at Universitas Negeri Makassar i.e. millennial students. • This data provides benefits for the Ministry of Education and Culture, Students, and Teachers in Indonesia. This data can be used by teachers, students, and policy makers in the field of education to carry out the education system of the industrial revolution 4.0 and how to overcome the problems regarding learning of the industrial revolution 4.0 in rural areas because there are still students who do not yet know and have used the internet or the like. • Future research might consider the use of other media whether it is suitable for use in learning the industrial revolution 4.0.

## 1 Data description

**Fig. 1:**
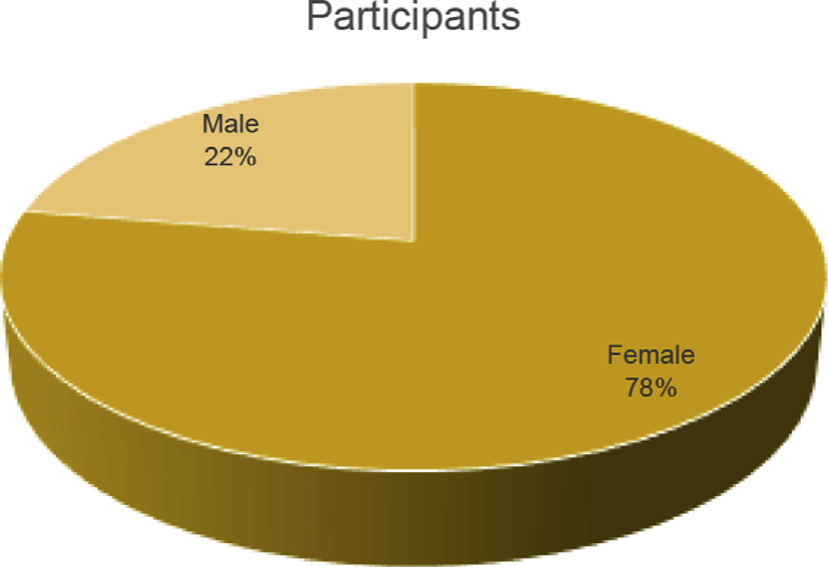
The sex of participants.

**Fig. 2:**
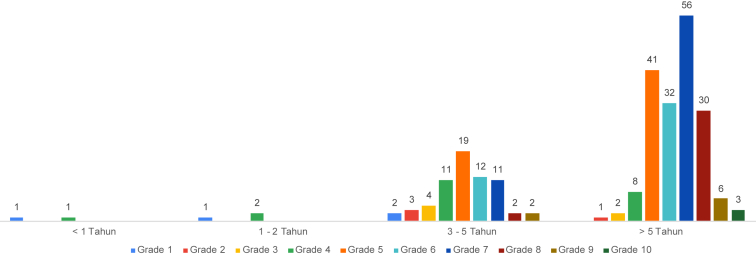
Participant computer's level of knowledge and long time using the internet.

From the questionnaire data, there are 250 participants with 194 females (78%) and 56 males (22%) (Fig. 1). Also, we can be shown the computer's level of knowledge and duration using the internet (Table 1 and Fig. 2).

**Table 1:** Computer's level of knowledge and long time using the internet of participant.

Level of Knowledge	Long Time Using Internet				Total
	<1 Year	1–2 Year	3–5 Year	>5 Year	
Grade 1	1	1	2	–	4
Grade 2	–	–	3	1	4
Grade 3	–	–	4	2	6
Grade 4	1	2	11	8	22
Grade 5	–	–	19	41	60
Grade 6	–	–	12	32	44
Grade 7	–	–	11	56	67
Grade 8	–	–	2	30	32
Grade 9	–	–	2	6	8
Grade 10	–	–	–	3	3
Total	2	3	66	179	250

Source: Field Survey, 2019.

The length of time students learn about the internet is categorized into 4 categories: (1) under 1 year, (2) 1–2 years, (3) 3–5 years, (4) over 5 years. From Table 1 and Fig. 2 it can be shown that students who have known the internet for more than 3 years have better computer skills compared to students who have known computers for a maximum of 2 years. From this sample, we can conclude that the longer a student is familiar with or knows the internet, the higher the level of computer knowledge is specific to FMIPA UNM students. This is because the majority of those who pass the selection into FMIPA UNM are derived from the interest of the Natural Sciences at High School. And of course this is different if it is done at the Faculty of Engineering where the dominant students come from the Vocational High School which certainly has a computer base.

**Fig. 3:**
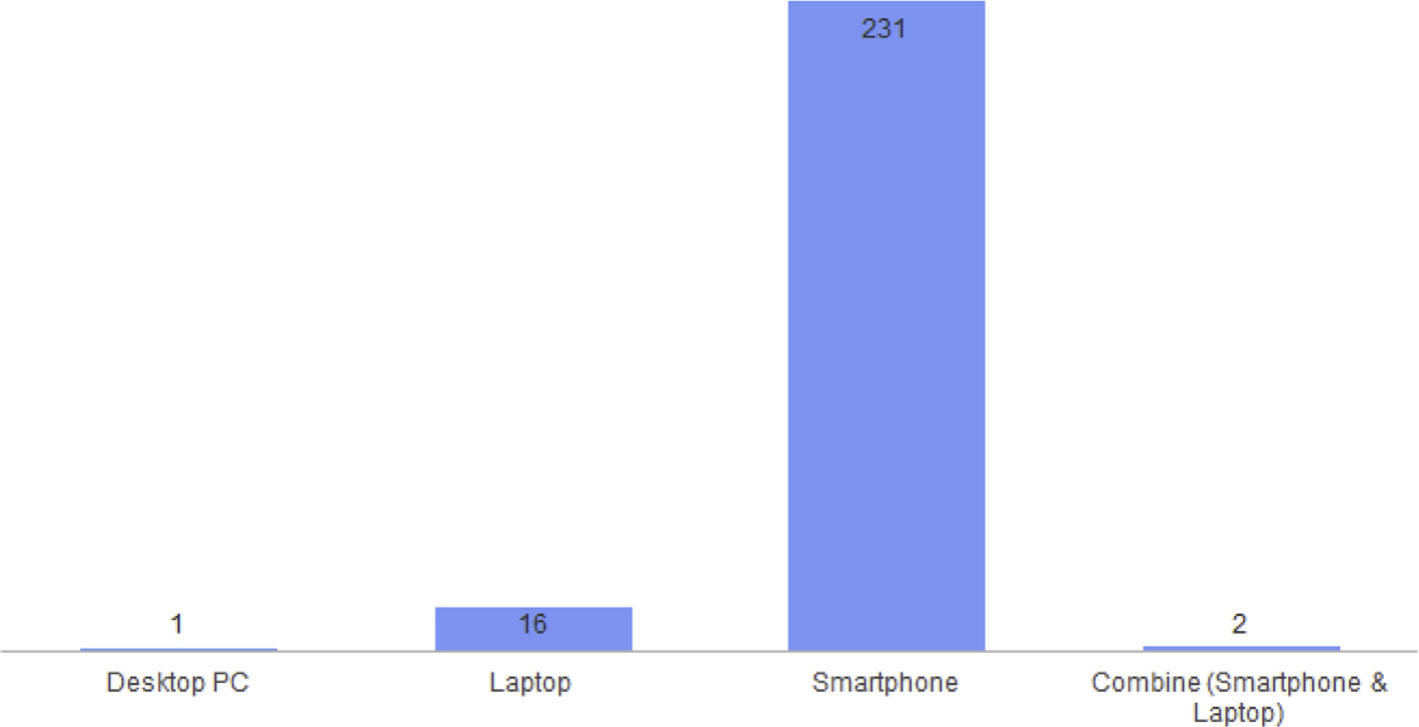
The devices often use to surf of participants.

**Fig. 4:**
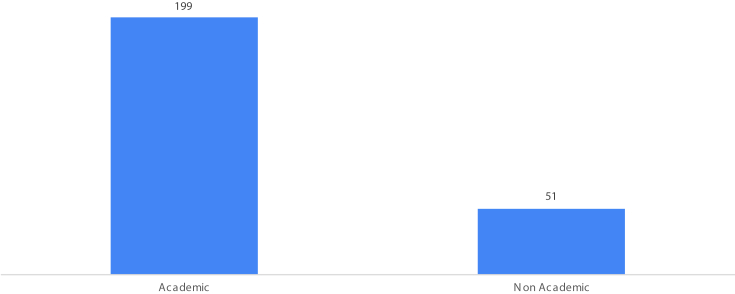
Things that are accessed by students when surfing the internet.

Apart from the ability of computers and duration using the internet, we can also see what devices are often used by students in accessing the internet (Fig. 3) and whether what is accessed is related to academic or non-academic (Fig. 4).

From Fig. 3, the students usually using Desktop PC, Laptop, Smartphone, and combine (Smartphone and Laptop) to surf internet. Fig. 3 show that students prefer to use their smartphone compared to other devices. This is in accordance with the results of the response, as many as 230 students (92%) are often used to surf compared to laptops, 16 students (6.4%) and 2.6% for other devices. This could be due to the ease of obtaining a smartphone compared to other devices (laptops), in addition to low prices, smartphones are also lightweight and easy to carry everywhere. This is in accordance with the opinion of Schindler, Burkholder, Morad, et al. said that many people rely more on smartphones for Internet access than more expensive devices such as laptops and tablets [1].

Fig. 4 show the 250 students who access the internet, around 51 students (20.4%) do not use the internet for academic purposes but are used for non-academic purposes such as social media or other news. This result suitable with the study of [2] that the highest internet use for academic purposes is students in social sciences, agriculture, and computer sciences, in FMIPA UNM, the dominant of students from the department of sciences (mathematics, biology, chemistry, geography, natural science) education.

Based on Table 2, the majority of students (>75%) agree that the use of online learning media (heutagogy models) can increase creativity, fun, willingness/motivation, and ability in the learning process. They can study anywhere and anytime without any limitations on space and time. This is in line with opinion Arkorful and Abaidoo which states that the use of technological tools enables students to learn anytime and anywhere, involves training, delivery of knowledge, and motivates students to interact with each other, and exchange and respect different points of view, and facilitate communication and improving relationships that support learning [3]. And the opinion of Sun and Chen that online learning will stimulate ongoing discussion about effective strategies that can increase the success of universities and faculties in the transition to teaching online and can help to improve higher education [4]. In the other case, there are several students disagree with online learning with reason e.g. learning online is indeed very easy to get information on the internet, but we also know what are the effects of the impact if it continues to use gadgets for learning media such as eye fatigue that can cause nearsightedness or farsightedness. The key concept in heutagogy is that of double-loop learning and self-reflection [5], so for this case, temporary, the heutagogy not fully online in Indonesia but blended learning.

**Table 2:** Rated of opinion/perception (%) of students about the online learning.

Questions of the Questionnaire	SA (%)	A (%)	D (%)	SD (%)
Do you agree that the learning system needs to keep up with the times?	56,00	42,80	0,80	0,40
Do you agree that current learning needs to follow a learning system that leads to the Industrial Revolution 4.0?	33,20	65,20	1,20	0,40
Do you agree with the online learning system?	23,60	66,80	8,40	1,20
Do you agree with the Heutagogy Learning system?	22,00	59,60	17,20	1,20
Do you agree that using the Heutagogy approach makes it possible to improve the learning process of students?	15,60	68,00	16,00	0,40
Do you agree if the teacher becomes your learning facilitator or consultant and not your learning companion?	19,20	58,40	22,40	0,00
Do you agree that using technology (ICT) will save teachers and students time?	20,80	69,60	8,40	1,20
Do you agree that using technology (ICT) in teaching and learning makes it easy?	23,20	70,80	5,60	0,40
Are you interested in learning if in learning to use ICT as a learning media?	21,20	73,20	5,20	0,40
Do you agree that the use of ICTs in learning attracts my attention so that it fosters learning motivation	17,20	74,00	8,40	0,40
Do you agree that the learning process is fun if in learning to use ICT?	13,60	75,60	10,00	0,80
Do you agree that the use of ICT can increase your creativity?	20,40	70,80	8,40	0,40
Do you agree that Learning becomes more real by utilizing ICT?	13,60	75,20	10,40	0,80
Do you agree that by using ICT, you are able to get the job done quickly?	23,20	71,60	5,20	0,00
Do you agree that with ICT, you can access information without being limited by distance, space and time, it can be anywhere and anytime?	50,80	47,60	1,60	0,00

Source: Field Survey, 2019.

We can be concluded that based on the data, the education policy makers can make a policy about the online learning with by paying attention to the situation of students both in terms of physical and financial abilities and of course it is also necessary to prepare facilities and infrastructure to support this online learning.

## 2 Experimental design, materials, and methods

This data adopted a descriptive survey design to see students' perceptions of online learning at FMIPA UNM. The descriptive survey design method is an efficient approach in gathering data about perceptions from a population sample.

### 2.1 Population

The population in this survey were students of FMIPA UNM who were active in academic information systems on Academic Year 2019. This research targets the representation of students from seventh departments in FMIPA UNM.

### 2.2 Sample

The sampling technique is carried out randomly from the population to be studied and ensures the representation of each department (purposive sampling) [6]. Researchers used online questionnaires to collect data for this survey. Researchers use online questionnaires because besides saving costs, it also makes it easier in the process of collecting and tabulating data. The first stage, the researchers conducted a trial on several UNM Postgraduate Program students (not included in the study sample) to ensure that there were no obstacles in using this online questionnaire. Data validation was used in filling out this questionnaire in the hope that no “mysterious” data would become research data. This validation is done by conducting a crosscheck directly about student data to the campus academic information system using NIM (student number), this validation will carry out the process of matching personal data input. If the data entered is not in accordance with academic information system data, then the data will read Not Available (NA) and the data will not be included in the data analysis. Respondents in this survey were given a time to fill in from October 14, 2019 to October 26, 2019 and there are 250 valid participants. At the end of time, the filling will be closed and the researcher will take the data for analysis.
